# Lipidomic and transcriptomic analysis and its therapeutic implications in Chinese Kazakh patients with esophageal squamous cell carcinoma

**DOI:** 10.1186/s12885-025-14858-7

**Published:** 2025-11-03

**Authors:** Qingchao Sun, Ruixue Liu, Haiping Zhang, Liang Zong, Tian Li, Liwei Zhang

**Affiliations:** 1 Department of Thoracic Surgery, The First Affiliated Hospital of Xinjiang Medical University, Xinshi District, 137 Liyushan South Road, Urumqi, Xinjiang 830000 China; 2 Department of General Surgery, The Fourth Affiliated Hospital of Xinjiang Medical University, Urumqi, 830000 China; 3 Institute of Integrative Medicine of Acute Abdominal Diseases, Tianjin Nankai Hospital, Tianjin Medical University, 8 Changjiang Avenue, Tianjin, 300100 China

**Keywords:** Esophageal squamous carcinoma, Kazakhs, Lipid metabolism, Lipidomics, AMP-activated protein kinase

## Abstract

**Objective:**

To analyze the lipidomic profile of ESCC patients, link changes in cancer lipid metabolism to gene expression changes, and provide new insights into the diagnosis and treatment of ESCC patients in the Kazakh Xinjiang ethnic group.

**Methods:**

By integrating the lipidome and transcriptome results, genes related to differential lipid metabolites in Kazakh ESCC patients were identified, and the effects of the key gene AMPK on lipid metabolism in ESCC cells were investigated by ultra-performance liquid chromatography/tandem mass spectrometry (UPLC‒MS/MS).

**Result:**

Through absolute lipid quantification analysis of two serum samples, 13 classes of lipids were detected, with triglycerides (TAGs) being the most abundant. Phosphatidylcholine (LPC), phosphatidylethanolamine (PE), and ceramide (Cer) were the lipid categories with significant differences between the two groups. Transcriptome analysis revealed that genes related to fatty acid synthesis, carnitine biosynthesis, and other lipid metabolism pathways were enriched in the tumor tissue. Integrative analysis of the two groups suggested that fatty acid synthesis, fatty acid metabolism, lipid degradation, cholesterol metabolism, and the AMPK signaling pathway were enriched in tumor tissue. UPLC‒MS/MS was used to perform targeted lipidomic analysis of AMPK-knockdown esophageal squamous cell carcinoma cells, suggesting that AMPK may be involved in the reprogramming of lipid metabolism in Kazakh ESCC patients.

**Conclusions:**

Lipid metabolic reprogramming occurs in the tumor tissue of Kazakh ESCC patients, and there is a correlation between AMPK activity and lipid metabolism, which suggests a potential therapeutic target for the treatment of Kazakh ESCC.

**Supplementary Information:**

The online version contains supplementary material available at 10.1186/s12885-025-14858-7.

## Introduction

Esophageal cancer (EC) [1–3] ranks sixth among cancers in terms of global prevalence, has substantial mortality [4], and ranks sixth in incidence and fourth in mortality in China. A mean of approximately 291,000 patients are diagnosed per year, with esophageal squamous cell carcinoma (ESCC) being the predominant histological type [5]. There are substantial geographical variations in ESCC incidence, with the Kazakh population of northern Xinjiang experiencing a higher incidence than the Han population or other ethnic groups. ESCC mortality remains at 68.88/100,000 for Kazakh populations despite treatment advances [6]. This region exhibits significant variations among its geographical areas and ethnic groups, pointing to unique risk factors for ESCC. While common high-risk behaviors like smoking and alcohol consumption are present, the unusual male-to-female ratio (1.2:1.0) and younger age of onset suggest additional early-life risk factors shared by both sexes but distinct from those in other high-risk areas, such as LinXian in China. These include consuming very hot, salted tea boiled with milk, a diet high in meat (especially salted, dried, or smoked) and dairy products, and low intake of fresh fruits and vegetables. These findings in northwestern China highlight the need to consider often-overlooked factors when studying ESCC risk in other regions, particularly Europe [7]. Hence, further research is needed to illuminate ESCC pathogenesis and identify biomarkers to improve treatment outcomes in the Kazakh population.

Lipid metabolism remains pivotal for human beings [8, 9]. Bioactive lipids influence signaling, with consequences for cell survival, proliferation and death, which impact tumor evolution, progression and metastasis [10]. Recent studies have confirmed that the levels of glycerophospholipids, especially those of phosphatidylcholine (PCs) and phosphatidylethanolamine (PEs), are decreased in Han Chinese patients with ESCC [11]. However, studies on the overall lipid metabolism levels in the serum samples of ESCC patients of the Kazakh ethnic group are rather limited. We performed nontargeted metabolomics on Kazakh ESCC patient blood samples in Xinjiang. Oleic acid and LysoPC (15:0) may promote tumor progression. Lipid types and contents could indicate ESCC trends [12]. Lipidomics is a branch of metabolomics that focuses on the comprehensive analysis of lipids (fats and fat-like molecules) in biological systems. It involves the identification, quantification, and characterization of lipid species and their interactions within cells, tissues, or biofluids (e.g., blood or plasma). Targeted quantitative lipidomics is more sensitive and specific than traditional lipidomics. It can accurately detect low-abundance lipids and perform high-throughput, precise analysis in complex samples [13, 14]. Transcriptomics is the large-scale study of RNA molecules (the *transcriptome*) in a cell, tissue, or organism at a specific time. It provides insights into gene expression patterns, regulatory mechanisms, and functional genomics [15]. Integration analysis of lipidomics and transcriptomics can provide a deeper understanding of tumor pathogenesis. For example, it can reveal changes in lipase expression and differences in lipid metabolism in pancreatic cancer [16].

**Fig. 1:**
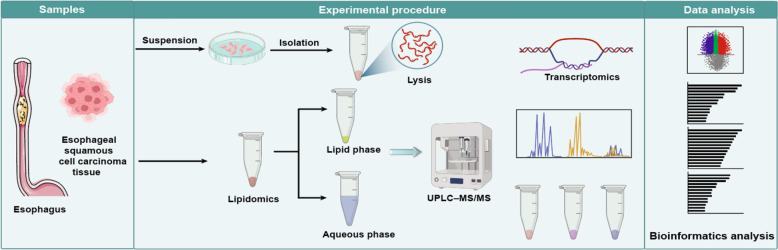
Schematic diagram of the integrated lipidomic and transcriptomic analysis of esophageal squamous cell carcinoma patients in the Kazakh ethnic group

Previous studies have had limitations in detecting lipid types and analyzing low-abundance phospholipids and sphingolipids. This study used targeted lipidomics for evaluating metabolic changes in esophageal squamous cell carcinoma. Transcriptome analysis revealed tumor-specific gene expression changes. Metabolic and transcriptome data were integrated to reveal dysregulated pathways (Fig. 1). Potential biomarkers for ESCC diagnosis and prognosis were explored.

## Materials and methods

### Research individuals

Thirty Kazakh patients who were diagnosed with ESCC and underwent surgery at the Thoracic Surgery Department of Xinjiang Medical University between Jul 2019 and Dec 2022 and 30 matched Kazakh controls were enrolled (Table S1). Inclusion criteria for the ESCC group:1) Kazakh patients who underwent radical surgery for esophageal cancer at the First Affiliated Hospital of Xinjiang Medical University. 2) Kazakh patients who underwent radical surgery for esophageal cancer at the First Affiliated Hospital of Xinjiang Medical University.3) Patients without overt metabolic diseases, including hypertension, diabetes, thyroid disorders, or rheumatic immune diseases. The exclusion criteria for the ESCC group:1) Patients who had received anti-tumor treatments (chemotherapy, radiotherapy, or traditional Chinese medicine) prior to specimen collection. 2) Those who have been taking anticoagulant drugs, weight-loss drugs, health care drugs, traditional Chinese (herbal) medicines and other drugs that may affect metabolism for a long time; 3) Patients with concurrent other malignant tumors; 4) Long-term vegetarians or unclear pathological stages. The inclusion criteria for the control group:1) The healthy Kazakhs who had undergone physical examination in the First Affiliated Hospital of Xinjiang Medical University showed no obvious abnormality. 2) There are no obvious metabolic diseases. The exclusion criteria for the control group were as follows:1) Menstruating women; 2) Pregnant/lactating women; 3) Metabolic diseases or long-term vegetarianism [12]. Serum samples were collected for lipidomic analysis. Tumor and adjacent normal tissues (≥ 5 cm from tumor lesions) from three randomly selected patients with ESCC were selected for transcriptomic analysis. Three patients were chosen to provide a representative view of the cohort and to allow statistical analysis to be performed. No participant had a history of radiotherapy, chemotherapy or metabolic disease prior to specimen collection.

### Serum sample collection and UPLC‒MS/MS analysis

Venous blood (5 mL) was collected from all participants on the morning of day 2 after admission or following an 8-h fast. The blood was stored at 4 °C for 2 h, centrifuged at 3000 × g for 15 min, and the serum was frozen at −80 °C. UPLC‒MS/MS was performed by the Metabolic Biology Company (Shanghai, China) [17]. UPLC‒MS can detect precise lipid molecular weights and structural fragments via mass spectrometry and tandem techniques for accurate lipid quantification. It also allows relatively accurate lipid quantification via standards or internal standards [18].

The target compounds were separated via liquid column chromatography via ultra-performance liquid chromatography (SCIEX Exion LC; Framingham, USA) with phase A consisting of 40% and 60% water and an acetonitrile solution containing 10 mmol/L ammonium acetate and phase B consisting of 10% and 90% acetonitrile and an isopropanol solution containing 10 mmol/L ammonium acetate. The mobile phase flow rate was 0.3 mL/min; the column temperature was 45 °C; and the plate temperature was 6 °C. A sample volume of 2 μL was injected. The AB Sciex QTrap 6500 mass spectrometer parameters were as follows: IonSpray voltage: + 5500/−4500 V; curtain gas: 40 psi; temperature: 350 °C; ion source gas 1: 50 psi; ion source gas 2: 50 psi; and DP: ± 80 V.

### Data processing and analysis

BioBud (v2.0.7; Shanghai, China) was used for data collection, summary, normalization and filtration. The absolute lipid content was calculated relative to the lipid internal standard by correlating the peak area with the actual concentration of a similar lipid internal standard (Table S2).

Multivariate statistical analysis of the UPLC‒MS/MS lipid profile data for Kazakh patients with ESCC and controls was performed via R software. Principal component analysis (PCA) and orthogonal partial least squares discrimination analysis (OPLS-DA) were conducted to achieve multidimensional statistical analysis. A PCA model was used to reduce data dimensionality and evaluate trend changes in lipid metabolism between the two groups. OPLS-DA was used to distinguish serum lipid profile differences between the two groups with variable importance projection (VIP) to determine the significance of each variable in the OPLS-DA model. The validity of the model was assessed via R2Y (the ability of the model to explain the categorical variable Y) and Q2 (the predictability of the model) following cross-validation. A permutation test was performed to generate various random Q2 values through multiple random changes in the permutation order of the categorical variable, Y, to test model validity.

Values of *p* < 0.05 from Student's t test and *VIP* > 1 from the first principal component of the OPLS-DA model were considered to indicate statistical significance. A volcano plot was generated to visualize the differentially expressed metabolites, and the degrees of change and metabolite classification were identified.

### Transcriptomic analysis

From the 30 patients with esophageal squamous cell carcinoma included in the study, 3 cases of cancer and adjacent tissues were randomly selected for transcriptome sequencing. All tumor samples were subjected to cryostat sectioning and histological examination to ensure their suitability for RNA extraction. The tumor cell content in each sample was visually assessed by a pathologist and confirmed via hematoxylin and eosin (H&E) staining. On the basis of the histological evaluation, the percentage of tumor cells in each sample was approximately 80%, which is sufficient for RNA extraction and downstream analyses. Total RNA was extracted via an RNA extraction kit (Invitrogen) and quantified via a NanoDrop ND-2000 spectrophotometer (Thermo Fisher Scientific, Waltham, MA, USA), and integrity was assessed via an Agilent Bioanalyzer 2100 (Agilent Technologies, USA). RNA-Seq libraries were prepared and sequenced with the Illumina HiSeq 2000 platform at Shanghai Biotechnology Co., Ltd. (Shanghai, China).

### Data acquisition

Array images were analyzed, and raw data were obtained via feature extraction software (version 10.7.1.1; Agilent Technologies) and analyzed via GeneSyring (version 13.1; Agilent Technologies). The raw data were normalized via a quantile algorithm, and probes flagged as"detected"under all the conditions were selected for further analysis. Differentially expressed genes (DEGs) were identified on the basis of a |fold change|≥ 2.0 and a *p* value ≤ 0.05. Gene Ontology (GO) and Kyoto Encyclopedia of Genes and Genomes (KEGG) analyses were conducted to assign functions to the differentially expressed mRNAs. Hierarchical clustering was conducted to identify gene expression patterns. The RNA-seq data were uploaded to the Gene Expression Omnibus database (GSE253171).

### Integration of metabolomics and transcriptomics data

Pearson correlation analysis was conducted on the differential lipids and lipid-related genes via the"corr.test"function in the"Psych"package of R language. The metabolite data from metabolomics and the gene expression data from transcriptomics were subsequently integrated and mapped to the KEGG database for pathway annotation. By collating all the pathway information related to species mapping, detailed pathway annotation results were obtained. Finally, the significance level of gene and metabolite enrichment in each pathway was calculated on the basis of Fisher's exact test method in statistics [16].

### Real-time quantitative PCR

cDNA was reverse transcribed from total RNA via the Revert Aid First Strand cDNA Synthesis Kit (Thermo Fisher Scientific, Inc.), following the manufacturer’s instructions. Real-time PCR was performed on a real-time PCR instrument (7500; Thermo Fisher Scientific) via a SYBR Green Master Mix kit (QIAGEN, Germany). The primers were designed via Primer 5.0 software and synthesized by Shanghai Sangon Bioengineering Co., Ltd. (Table S3). The PCR volume was 20 μL, and 40 cycles of 95 °C for 2 min, 95 °C for 15 s and 58 °C for 34 s were conducted in accordance with the manufacturer’s instructions. Relative mRNA expression levels were quantified by calculating the 2^−ΔΔCT^ value with *GAPDH* as a reference. Each test was conducted in triplicate.

### Cell culture

The EC cell lines KYSE150 and TE-1 were acquired from the American Type Culture Collection (ATCC). We performed short tandem repeat (STR) profiling and monitored the cell lines for contamination and mycoplasma infection throughout the test period via mycoplasma-specific PCR. The cells were cultured in Roswell Park Memorial Institute (RPMI) 1640 medium supplemented with 10% fetal bovine serum (FBS), 100 U/mL penicillin and streptomycin (BI, Cromwell, CT, USA) at 37 °C in 5% CO_2_ and passaged at 80% confluence. The cells that exhibited robust growth and reached confluence (80–90%) were selected for subsequent experiments.

### Western blotting

The cells were collected and lysed on ice in western blotting lysis buffer (Beyotime, China) to extract proteins, and the protein concentration was assessed via a BCA protein quantification kit (Solarbio, China). Proteins were separated by sodium dodecyl sulfate‒polyacrylamide gel electrophoresis and transferred to 0.45-μm polyvinylidene difluoride membranes (Millipore, USA; 300 mA, 90 min) at 4 °C overnight. The membranes were washed three times with TBST, blocked with 5% skim milk powder for 2 h, and incubated overnight at 4 °C with an anti-AMPK antibody (1:1000, Abcam) or rabbit anti β-actin (1:2000, Proteintech). The membranes were incubated for 1 h with secondary antibody (1:1000; Proteintech), and the proteins were visualized with enhanced chemiluminescence (ECL) reagent (Solarbio, China) and ImageJ software. The relative expression of the AMPK proteins [19, 20] calculated by the ratio of the gray value of the target protein to that of the reference protein (β-actin, 1:1000; Proteintech, China).

#### UPLC‒MS/MS lipid metabolism analysis

The interference vector for lentiviral packaging was constructed by Shanghai Hanheng Technology Co. Ltd. KYSE150 cells were inoculated into six-well culture plates at a density of 1 × 10^6^ cells/well and infected at 30–50% confluence with a mixture of lentivirus and 6 μg/mL polybrene for 72 h at 37 °C. The culture medium was replaced with fresh medium containing 10% FBS, and the cells were incubated for 6–8 days before screening with 2 μg/mL puromycin. The shRNA knockdown efficiency was verified via western blotting, and shRNA3-AMPK and shRNA-NC cells were harvested, washed with precooled PBS, and trypsinized. The cells (1 × 10^7^) were resuspended in PBS and centrifuged in a rotor precooled to −20 °C at 2500 × g for 5 min at 4 °C. The supernatant was discarded, and the cells were flash-frozen in liquid nitrogen for 30 s and stored at −80 °C. The cell lysate was preserved on dry ice at Shanghai Achu Biotechnology Co., Ltd., and UPLC‒MS/MS was performed as previously described.

#### Statistical analysis

Statistical analysis was performed via SPSS 26.0 (IBM, Armonk, NY, United States), GraphPad Prism version 8.4.0 (GraphPad, San Diego, CA) and R 4.0.3. Continuous variables with a normal distribution are expressed as the mean ± standard error (SEM), and nonnormally distributed data are expressed as the median and interquartile range. Categorical variables are expressed as proportions and quantities. A value of *P* < 0.05 was considered statistically significant.

## Results

### Baseline data and blood biochemical indexes

Serum total cholesterol, protein and albumin levels were significantly lower in patients with ESCC than in controls (*P* < 0.0001; *P* < 0.0001; *P* < 0.0001;). No differences were found in age, sex, triglycerides, total bile acid, HDL-C, or low-density lipoprotein cholesterol between the two groups (Table 1).

**Fig. 2:**
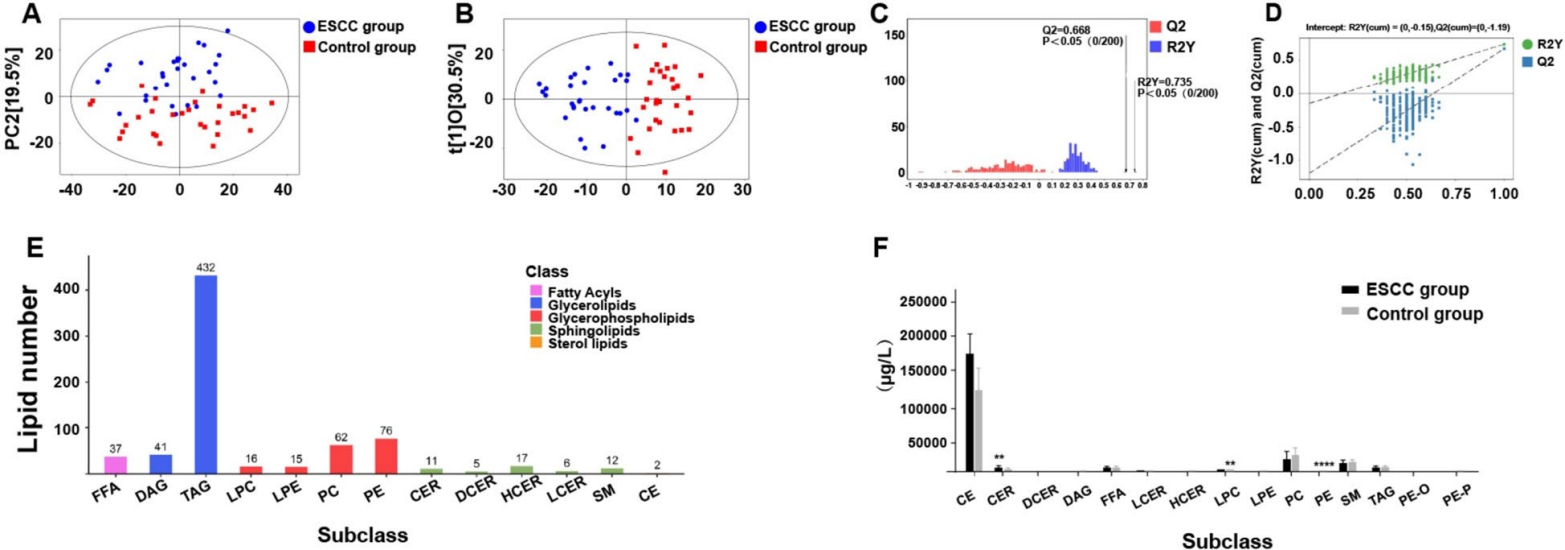
Multivariate analysis of the serum lipid compositions and contents in the ESCC and normal control groups. **A** Scatter plot depicting principal component analysis of the serum metabolomics data from the ESCC and normal control groups. **B** Scatter plot illustrating OPLS-DA of serum metabolomics data from the ESCC and normal control groups. **C** Validation diagram of the OPLS-DA model, R2X = 0.49, R2Y = 0.735, and Q2 = 0.668, with statistical significance at *P* < 0.05. **D** Correlation coefficients of R2Y and Q2. The green dots represent R2Y values obtained from the permutation test, whereas the blue square dots depict Q2 values from the same test. The dashed lines depict regression lines for R2Y and Q2, respectively. **E** Global lipid subclasses and the corresponding lipid numbers in the serum samples of both groups. **F** Total quantification of each lipid subclass in the serum samples of both groups. ** represents* P* < 0.01, and **** represents* P* < 0.0001

The serum metabolomic data of patients with ESCC were log transformed with SIMCA software, and PCA and OPLS-DA were used to reduce data dimensionality and to identify differences in the serum metabolomic profiles. Abscissa PC1 and ordinate PC2 show scores of the first- and second-ranked principal components and suggest distinct differences between patients with ESCC and controls (Fig. 2A). The degree of intergroup and intersample variation was assessed via OPLS-DA, and the principal components indicated by the abscissa represent the former, and the orthogonal principal components indicated by the ordinate represent the latter (Fig. 2B). The validation results from the OPLS-DA model revealed that the explanatory power of the X variable was 0.49 and that of the Y variable was 0.735, with a predictive power (Q2) of 0.668. This suggests that the model utilized 49.0% of the original variables to account for 73.5% of the variation between the ESCC and control serum metabolomic data, with a predictive power of 66.8% (Fig. 2C). The predictive power of the model exceeded 50%, with the difference between R2 (Y) (the difference between the groups that could be attributed to the model) and Q2 being < 0.3. These findings suggest that the OPLS-DA model fit was good (Fig. 2D).

### Serum lipid composition and subclasses

Lipids were categorized into subclasses on the basis of head group. Biobud-v software was utilized for quantitative analysis of all target lipids. Thirteen lipid compounds were detected in total, with triglycerides being the most abundant (Fig. 2E). Lysophosphatidylcholine (LPC) and phosphatidylethanolamine (PE) were found to be significantly lower in patients with ESCC than in controls (*P* < 0.001; *P* < 0.001), and Cer was elevated (*P* < 0.01). The total free fatty acid (FFA), diacylglycerol (DAG), triacylglycerol (TAG), lysophosphatidyl ethanolamine (LPE), phosphatidyl choline (PC), dihydroceramide (DCER), hexosyl ceramide (HCER), lactosyl ceramide (LCER), sphingolipid (SM) and cholesteryl ester (CE) contents were not significantly greater in the ESCC group than in the control group (Fig. 2F). Ceramides are a type of sphingolipids, and their dysregulation contributes to the uncontrolled growth of cancer cells. Studies have shown that the elevation of intracellular Cer and sphingosine 1-phosphate (S1P) levels is mainly associated with the induction of cell cycle arrest, apoptosis and autophagy [21]. For instance, in breast cancer, increasing Cer levels by inhibiting the formation and/or accumulation of S1P is emerging as a promising target for suppressing tumor growth and overcoming drug resistance [22]. Lysophosphatidylcholine (LPC), a major component of oxidized low-density lipoprotein, can bind to G protein-coupled receptors and toll-like receptors, inducing the migration of lymphocytes and macrophages. Current research indicates that the concentration of LPC varies among different tumors and plays a significant role in tumor invasion, metastasis and prognosis [23]. Alterations in lipid profiles may reflect abnormal lipid signaling in the tumor microenvironment.

### Serum lipid chain length and degree of unsaturation

**Fig. 3:**
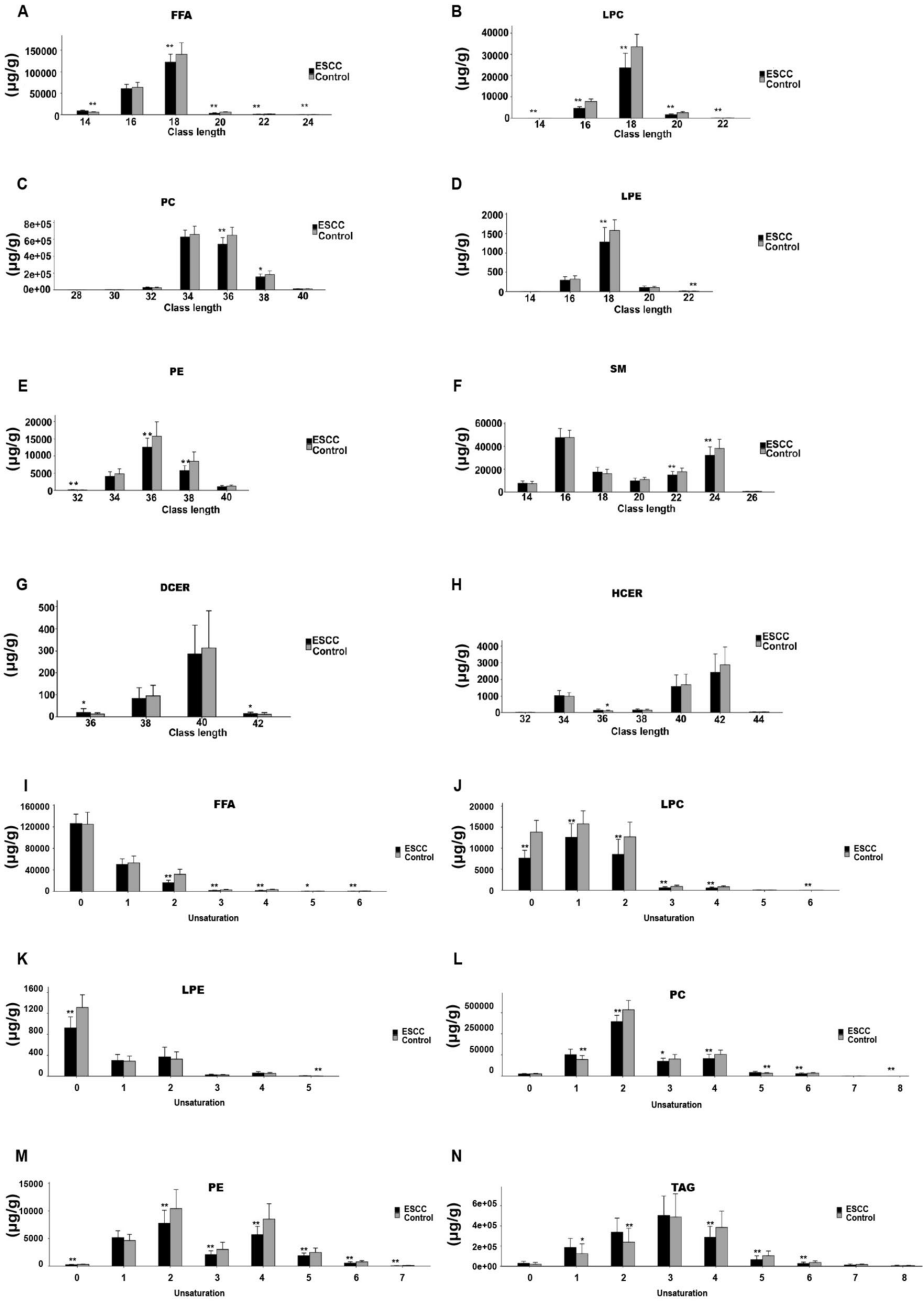
Concentrations of lipids with different carbon chain lengths, degrees of carbon chain unsaturation in total FFAs, and differences in each lipid compound. **A**–**H**: Concentrations of FFAs, LPC, PC, LPE, SM, PE, DCER, and HCER with various carbon chain lengths. **I**–**N** Concentrations of lipids with different degrees of unsaturation in FFAs, LPC, LPE, PC, PE, and TAG. * represents *P* < 0.05; ** represents *P* < 0.01)

A decreased content of FFAs with chain lengths of 18, 20, 22, and 24 was found in the ESCC group(*P* < 0.01;* P* < 0.01;* P* < 0.01;* P* < 0.01), and FFAs with a chain length of 14 were more abundant compared with controls (*P* < 0.01) (Fig. 3A). Compared with that in the control serum, the LPC subclasses in the ESCC serum had lower chain length diversity (*P* < 0.01) (Fig. 3B). In addition, PC lipids with chain lengths of 36 and 38 were reduced in patients with ESCC compared with controls (*P* < 0.01;* P* < 0.05) (Fig. 3C), and LPE levels were decreased, particularly those with chain lengths of 18 and 22 (*P* < 0.01;* P* < 0.01) (Fig. 3D). PEs of greater carbon chain length were decreased in ESCC serum (*P* < 0.01) (Fig. 3E), as were SM lipids of chain lengths 22 and 24 (*P* < 0.01;* P* < 0.01) (Fig. 3F). In contrast, higher DCER and HCER values of chain length 36 were observed in the tumor group than in the control group (*P* < 0.05;* P* < 0.05) (Fig. 3G and H). Most unsaturated FFA, LPC, LPE, PC, PE and TAG lipids were downregulated in the ESCC samples compared with the control samples (*P* < 0.05) (Fig. 3I–N), and the levels of other unsaturated lipid compounds were also significantly lower in the tumor group. In summary, alterations in the lipid profile related to chain length and degree of unsaturation were observed in Kazakh patients with ESCC.

### Screening of differential serum lipids

**Fig. 4:**
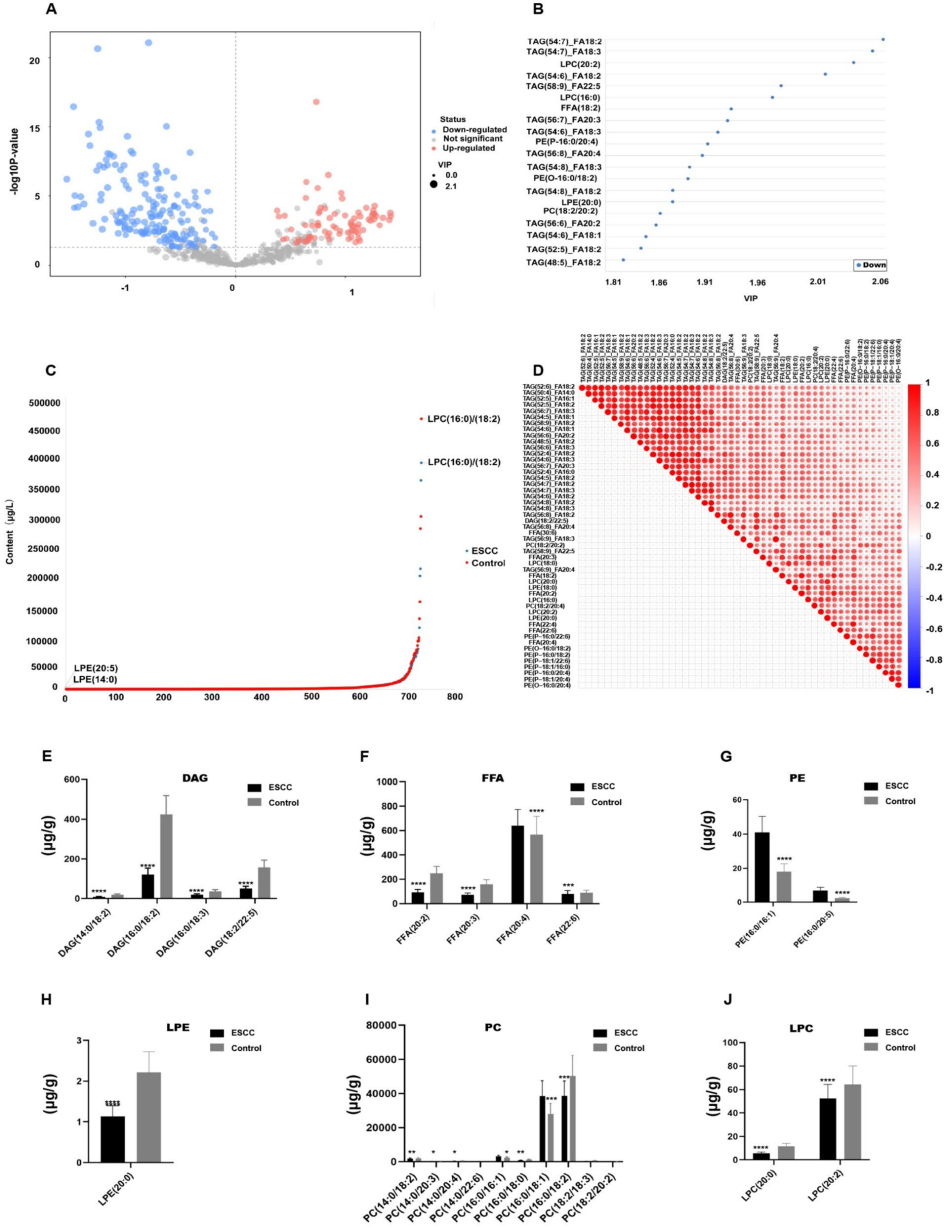
Differences in the expression of serum lipids and corresponding metabolite concentrations between the ESCC and normal control groups. **A** Volcano plot illustrating differential lipids. **B** The top 20 lipids with the highest VIP values in the OPLS-DA model. **C** Dynamic range analysis of the lipid content in the two groups. **D** Correlation analysis of the differentially expressed lipids in serum samples from the ESCC and normal control groups. **E**–**J** Concentrations of DAG, FFA, PE, LPE, PC, and LPC in the ESCC group and control group. *,* P* < 0.05; **,* P* < 0.01; ***, *P* < 0.001; ****, *P* < 0.0001

A total of 249 lipid metabolites were significantly different between patients with ESCC and controls, of which 79 were upregulated and 170 were downregulated in the ESCC group (Fig. 4A). The 20 lipids with the largest VIPs in each group, all of which were downregulated, were used to construct the OPLS-DA model (Fig. 4B). Pearson’s correlation analysis of the 50 top-ranked lipids with the highest VIPs revealed a significant positive correlation between several triglycerides and other lipids (Fig. 4C). Examination of the dynamic range of lipid content revealed that the content of PCs (16:0/18:2) was high in both groups. However, some differences were apparent, with LPE 14:0 being lowest in ESCC samples and LPE 20:5 being lowest in controls (Fig. 4D). DAG (14:0/18:2), (16:0/18:2), (16:0/18:3) and (18:2/22:5) were all downregulated (*P* < 0.001; *t* = 5.235, *P* < 0.0001; *P* < 0.001;* P* < 0.0001), with the 18:2/22:5 species showing a 0.37-fold decrease (Fig. 4E). FFA (20:2), (20:3), (20:4) and (22:6) were all downregulated (*P* < 0.0001;* P* < 0.0001;* P* < 0.0001;* P* < 0.0001), with the 20:4 species downregulated 0.36-fold (Fig. 4F). LPC (20:0), (20:2) and LPE (20:0)were all downregulated (*P* < 0.0001;* P* < 0.0001;* P* < 0.0001) (Fig. 4G-H).The differential abundances of PC (14:0/18:2), PC (14:0/20:3), PC (14:0/20:4), PC (14:0/22:6), PC (16:0/16:1), PC (16:0/18:0), PC (16:0/18:1), PC (16:0/18:2) are shown in Figs. 4I (*P* < 0.001;* P* < 0.01;* P* < 0.001;* P* < 0.0001; *P* < 0.0001;* P* < 0.0001; *P* < 0.0001). The glycerolipids PE (16:0/16:1) and PE (16:0/20:5) were upregulated 2.28- and 2.48-fold (*P* < 0.0001;* P* < 0.001), respectively, in the tumor group (Fig. 4J). KEGG analysis revealed that glycerophospholipid, arachidonic acid, linoleic acid, and choline metabolism were enriched in the lipidomic profile of Kazak patients with ESCC (Figure S1). Significantly altered lipid metabolites and pathways were found in patients with ESCC.

### DEGs between ESCC and adjacent normal tissues

**Fig. 5:**
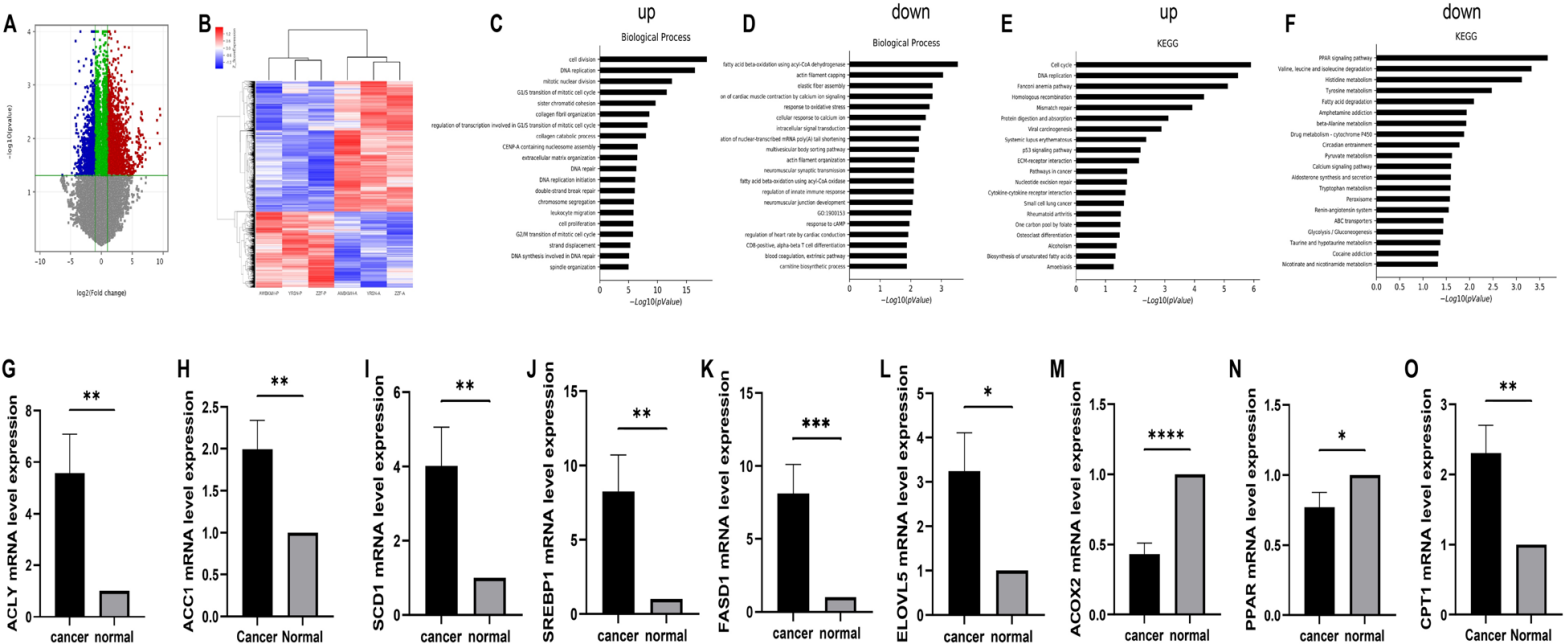
DEGs and expression of lipid metabolism-related genes between tumor and adjacent normal tissues of Kazakh patients with ESCC. A Volcano plot illustrating DEGs. B Heatmap generated from hierarchical cluster analysis. C–D GO analysis depicting the top 20 enriched functions of the upregulated (C) and downregulated DEGs (D). E–F KEGG analysis showing the top 20 enriched pathways of the upregulated (E) and downregulated DEGs (F). G–O RT‒qPCR analysis of the mRNA expression levels of ACLY, ACC1, SCD1, SREBPF1, FADS1, ELOVL5, PPAR, CPT1, and ACOX2 in ESCC and adjacent normal tissues. *N* = 30. * P < 0.05, ** P < 0.01, *** P < 0.001, and **** P < 0.0001

RNA-seq analysis of tumor and adjacent normal tissues revealed DEGs involved in lipid metabolism. In total, 4612 DEGs met the criteria of |fold change|≥ 2 and *p* < 0.05, including 3018 DEGs whose expression was upregulated and 1594 DEGs whose expression was downregulated in tumor tissues (Fig. 5A). Hierarchical cluster analysis suggested a clear distinction between ESCC and adjacent normal tissues on the basis of DEG profiles. Particularly clear differences were observed with the probes AWBKMH-P, YRSN-P, ZZF-P, AWBKMH-A, YRSN-A, and ZZF-A (Fig. 5B).

GO analysis revealed that the upregulated DEGs were associated primarily with “cell division,” “DNA replication,” and “mitotic nuclear division” (Fig. 5C). The downregulated DEGs were associated with “fatty acid beta-oxidation,” “actin filament capping,” “elastic fiber assembly,” and “carnitine biosynthesis process” (Fig. 5D). KEGG pathway analysis revealed that the upregulated DEGs were significantly enriched in “cell cycle,” “DNA replication,” and “unsaturated fatty acid biosynthesis pathway” (Fig. 5E). The downregulated DEGs were enriched in “PPAR signaling pathway,” “valine, leucine and isoleucine degradation,” “histidine metabolism,” and “fatty acid degradation” (Fig. 5F). Profiles of DEGs between Kazakh patients with ESCC and controls revealed considerable involvement of lipid metabolism.

### Expression of lipid metabolism-related genes

The mRNA levels of genes associated with unsaturated fatty acid synthesis, including ACLY, ACC1, SCD1, SREBP1, FADS1 and ELOVL5, were significantly greater in ESCC tissues than in adjacent normal tissues (*P* < 0.01;* P* < 0.01;* P* < 0.01;* P* < 0.01;* P* < 0.001;* P* < 0.05) (Fig. 5G–I), suggesting active unsaturated fatty acid synthesis and de novo lipogenesis in tumor tissues. An investigation of genes related to fatty acid β-oxidation*, s*uch as PPAR, CPT1, and ACOX2, revealed that ACOX2 and PPAR were decreased in ESCC tissues but that CPT1 was upregulated (*P* < 0.0001; *P* < 0.05; *P* < 0.05) (Fig. 5M–O). Thus, significant alterations in lipid metabolism-related genes were present in the ESCC tissues of Kazakh patients.

### Integrated analysis of the lipidomic and transcriptomic data

Integrated analysis of lipidomic data with paired DEG transcriptomic data revealed 93 DEGs related to lipidomics (Figure S2A). KEGG analysis revealed that the DEGs were enriched in “PPAR signaling pathway,” “biosynthesis of unsaturated fatty acid,” “regulation of lipolysis in adipocytes,” “cholesterol metabolism,” “glycerophospholipid metabolism,” and “AMPK signaling pathway” (Figure S2B). FADS1 and ELOVL5, which are involved in unsaturated fatty acid biosynthesis, and ACLY, which is involved in general lipid synthesis, were enriched. ACOX2, which is involved in adipocyte lipolysis regulation, and AMPKα2 (PPKAA2), which is involved in the AMPK signaling pathway, were also enriched. Higher FPKM values for FADS1, ELOVL5, and ACLY were detected in cancer tissues than in adjacent normal tissues (*P* < 0.01; *P* < 0.01;* P* < 0.01), suggesting that alterations in lipid metabolism in ESCC are mediated by altered expression of these genes (Figure S2C). The FPKM values of ACOX2 and PPKAA2 were lower in cancer tissues than in adjacent normal tissues (*P* < 0.05;* P* < 0.01), again indicating the potential involvement of these genes (Figure S2C).

### Role of AMPK in lipid metabolism

**Fig. 6:**
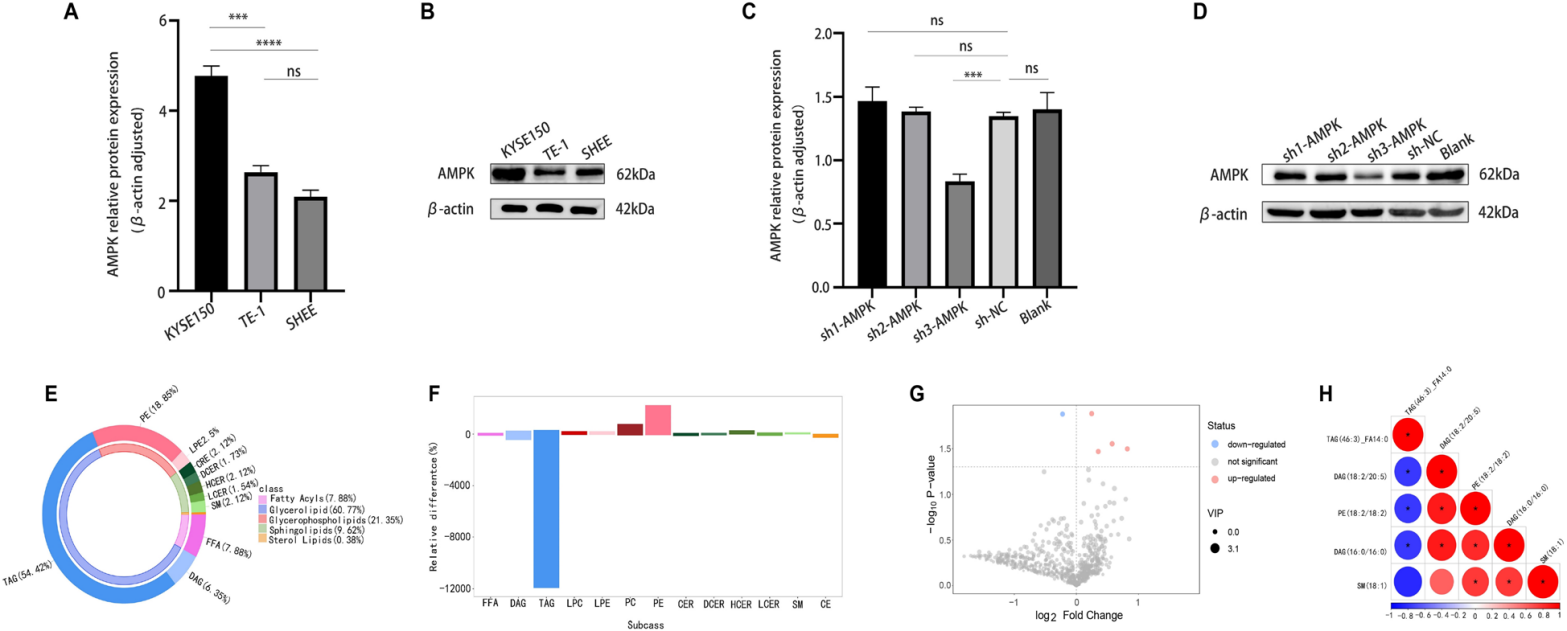
AMPK and its targeted lipids in esophageal cancer cells. A and B: Representative immunoblotting image (B) and quantification (A) of AMPK expression in KYSE150, TE-1, and SHEE cells. Statistical significance: *** P < 0.001, ****P < 0.0001; ns, no significant difference. C and D: Representative immunoblotting image (D) and quantification (C) of AMPK expression in KYSE150 cells infected with lentivirus carrying sh-AMPK or shNC. ***P < 0.001. E: Annular diagram illustrating lipid composition. F: Histogram presenting lipid levels in the sh3-AMPK group versus the shNC group. G: Volcano plot displaying the differentially expressed metabolites identified in the sh3-AMPK group versus the shNC group. H: Correlation thermogram of differentially expressed metabolites identified in the sh3-AMPK group versus the shNC group

The AMPK signaling pathway was associated with DEGs and metabolites, and AMPK expression in the EC cell lines KYSE150 and TE-1 and the normal esophageal cell line SHEE was compared. AMPK expression was greater in KYSE150 cells than in SHEE cells (*P* < 0.01), but no significant difference was detected in TE-1 cells (Fig. 6A and B). Lentiviral AMPK knockdown with sh-3-AMPK in KYSE150 cells was successful according to western blotting (*P* < 0.05) (Fig. 6C and D), and these cells were used for UPLC‒MS/MS analysis. Ten lipids, including fatty acids (7.88%), glycerides (60.77%), glyceride sphingolipids (21.35%), sphingolipids (9.62%), and sterol lipids (0.38%), were identified (Fig. 6E-F). Compared with those in shNC-transfected cells, five metabolites were significantly different in the sh3-AMPK group: four were upregulated(*P* < 0.05;* P* < 0.01;* P* < 0.05;* P* < 0.05), namely, DAG (16:0/16:0), DAG (18:2/20:5), PE (18:2/18:2), and SM (18:1), and one was downregulated, TAG (46:3)FA14:0 (*P* < 0.05) (Fig. 6G). Correlation analysis revealed a negative correlation between the downregulation of TAG (46:3) FA14:0 and the upregulation of DAG (16:0/16:0), DAG (18:2/20:5) and PE (18:2/18:2)(*P* < 0.05;* P* < 0.01; *P* < 0.05) (Fig. 6H). These findings suggest that AMPK is involved in the production of DAG (16:0/16:0), DAG (18:2/20:5), PE (18:2/18:2), SM (18:1), and TAG (46:3) FA14:0 in KYSE150 cells.

## Discussion

### Main interpretation

EC is a malignant tumor that has high mortality rates and has been linked to substantial alterations in lipid metabolism. Differences in the serum lipid subclass content, chain length, and degree of unsaturation between patients with ESCC and controls were found during the current study, with differential expression of genes implying increased rates of beta-oxidation in tumors. Integrated analysis demonstrated increased biosynthesis of unsaturated fatty acids, fatty acid metabolism, and AMPK signaling in ESCC tissues. UPLC–MS/MS analysis of AMPK-deficient ESCC cells suggested a role for this enzyme in mediating lipid metabolism. Our study highlights the significant alterations in lipid metabolism in patients with ESCC, which may lead to the discovery of novel therapeutic agents.

The upregulation of genes associated with lipid synthesis appears to be a general characteristic of tumor tissue and reflects the need for the generation of biomass. Inhibitors of lipid synthesis, including the ACLY inhibitor SB-204990 and ACC inhibitors (TOFA, soraphen A and ND646), have been developed as potential anticancer drugs, and phase I clinical trials of the FSCN inhibitor TVB-2640 have been conducted [24]. These previous studies indicate the anticancer potential of disrupting lipid synthesis. The findings of elevated ACLY and ACC1 in ESCC tissues in the present study indicate that these enzymes may be promising therapeutic targets for ESCC treatment. Reprogramming of lipid metabolism pathways in ESCC, including those associated with sphingolipids and glycerolipids, has been previously demonstrated, and the results of the present study revealed consistent upregulation of these pathways in Kazakh patients with ESCC. The Kazakh ethnic group has a high incidence of ESCC, and we previously reported elevated glycerolipid metabolism, particularly oleic and arachidonic acid metabolism, in patients with ESCC with lymph node metastasis [12], with implications for the identification of therapeutic targets. Patients with ESCC in the present study had elevated total triglycerides and lower long-chain fatty acids. Long-chain fatty acids and lipids with diverse saturations have previously been shown to promote tumor progression and metabolic reprogramming of immune cells within the TME [25]. Cancer cells must upregulate lipid production to accommodate the synthesis of membranes to support excessive cell proliferation. Differences in the saturation of membrane phospholipids have been suggested for tumor cells with an increased content of saturated fatty acids, increasing the resistance of phospholipids to peroxidation [26].

Many lipid alterations involved in membrane formation were identified in the current analyses.

GPs are the most common and abundant phospholipids in the body. Phosphatidylcholine (PC) is the most abundant glycerophospholipid in the membranes of eukaryotic cells and is associated with tumor cell proliferation and signal transduction [27]. The relative abundances of PC(20:5/15:0) and PC(22:6/13:0) were greater in ESCC patients than in healthy controls, whereas the level of PC(14:1/20:4) was lower in ESCC patients [11]. Studies have shown that PLA2 is highly active and is overexpressed in ovarian and breast cancers, which may lead to a decrease in PC levels in patients'serum [15]. LPC and LPE are metabolites of the membrane components PC and PE. LPCs with varying chain lengths and saturation levels differed between ESCC patients and controls, suggesting disordered PC metabolism in Kazakh ESCC patients. Previous studies have shown consistently dysregulated LPC levels in ESCC [28, 29], and LPE is known to inhibit phospholipase D (PLD) and influence tumor cell proliferation and migration [30]. Elevated serum LPE (16:0), (20:4) and (18:1) has previously been found in patients with ESCC [31], although higher levels of LPE (22:5) and (16:1) were found during the current work. These results demonstrated the potential for disturbed glycerophospholipid levels, especially LPC and LPE, as ESCC biomarkers.

The membrane components SM, (22:0), (22:1) and (24:0) were also decreased, and Cer (18:0/26:1) was increased in patients with ESCC. Cer (d18:0/24:0) and (d18:0/24:1) were previously reported to be increased in metastatic ESCC, and both Cer species inhibited metastasis in vivo [32, 33]. Ceramide is a biologically active sphingolipid that participates in mitochondria-mediated apoptosis. Cer promotes apoptosis by regulating the release of mitochondrial cytochrome C [33, 34], and ceramide directly regulates mitochondrial outer membrane permeabilization (MOMP)-mediated apoptosis [35]. Most studies thus far have shown that ceramides regulate apoptosis mainly through ceramide channel formation and the regulation of antiapoptotic Bcl2 family proteins (Bcl2 and Bcl-xL) and proapoptotic Bcl2 family proteins [36].SM also mediates autophagy by disrupting autophagosome organization, and its levels are mediated by the enzyme autophagy-related protein 9 A (ATG9A) [34, 37]. Recent cancer therapy approaches have focused on increasing ceramide biosynthesis to trigger programmed cell death. These findings suggest that SM may be a promising metabolite for treating ESCC metastasis.

AMPK is considered an intracellular energy sensor [37] and phosphorylates acetyl-CoA carboxylase 1 to inhibit fatty acid synthesis and stimulate fatty acid oxidation, reducing fatty acid accumulation in hepatocytes [38–40]. HMG CoA reductase, the rate-limiting enzyme for hepatic cholesterol synthesis, is also a target of AMPK [29, 41]. AMPK also has commensurate effects on gene transcription, phosphorylating Ser372 of the fatty acid regulator SREBP1 and suppressing its expression [39]. Tumor cells depend on lipid biosynthesis to fuel proliferation, and the downregulation of lipid synthetic pathways in response to the detection of low energy status by AMPK would generally be considered unfavorable to the tumor strategy. AMPK was upregulated in ESCC cells in the present study and may be involved in the metabolism of glycerides, glycerophosphatides, and sphingomatides; however, further work is necessary to identify the underlying mechanisms involved.

### Limitations

We acknowledge some limitations to the current study. First, we believe that AMPK is involved in lipid metabolism disorders in Kazakh ESCC patients. We propose large-scale longitudinal studies to validate our findings. These studies will use advanced multiomics platforms and standardized protocols. Second, the study focused on Kazakh-origin participants in China to minimize variables and investigate a population with high ESCC rates, but this limited participant eligibility. These findings may not apply to other ethnic groups and require further validation. Third, transcriptomic analysis was conducted on three randomly selected samples out of 30, which was sufficient for statistical analysis, but a larger sample size is needed for validation.

### Conclusion

In summary, the lipid profiles of Kazakh patients with ESCC were significantly altered relative to those of controls. AMPK was found to affect lipid metabolism in ESCC cell lines, an effect verified by AMPK knockdown. We conclude that AMPK is involved in the altered lipid profile observed in patients with ESCC and reveals potential therapeutic targets. These findings highlight the importance of lipid metabolism in tumor progression and offer insights into lipid reprogramming during ESCC.

## Supplementary Information

Supplementary file 1. 

## Data Availability

Data are available from corresponding author upon reasonable requests.

## Abbreviations

ACLY: ATP citrate lyase
AMPK: ATP-activated protein kinase
CE: Cholesteryl ester
Cer: Ceramide
DAG: Diacylglycerol
DCER: Dihydroceramide
DEGs: Differentially expressed genes
EC: Esophageal cancer
ESCC: Esophageal squamous cell carcinoma
FFA: Free fatty acid
GEO: Gene Expression Omnibus
GO: Gene Ontology
HCER: Hexosyl ceramide
KEGG: Kyoto Encyclopedia of Genes and Genomes
LCER: Lactosyl ceramide
LPC: Lysophosphatidylcholine
LPE: Lysophosphatidyl ethanolmine
OPLS-DA: Orthogonal partial least squares discrimination analysis
PC: Phosphatidylcholine
PCA: Principal component analysis
PE: Phosphatidylethanolamine
PVDF: Polyvinylidene difluoride
SDS‒PAGE: Sulfate polyacrylamide gel electrophoresis
SM: Sphingolipid
TAG: Triacyglycerol
UPLC–MS/MS: Ultra-performance liquid chromatography/tandem mass spectrometry
VIP: Variable importance projection
