# Supplementary material for: Lipidomic and transcriptomic analysis and its therapeutic implications in Chinese Kazakh patients with esophageal squamous cell carcinoma
Source: BMC Cancer. 2025 Nov 3;25:1696. doi: 10.1186/s12885-025-14858-7 (PMC12581371; doi:10.1186/s12885-025-14858-7)
Supplement: Supplementary file 1 [file 12885_2025_14858_MOESM1_ESM.docx]

**Figure S1. KEGG enrichment analysis of the differential lipid metabolites in the ESCC group and the control group.**

**
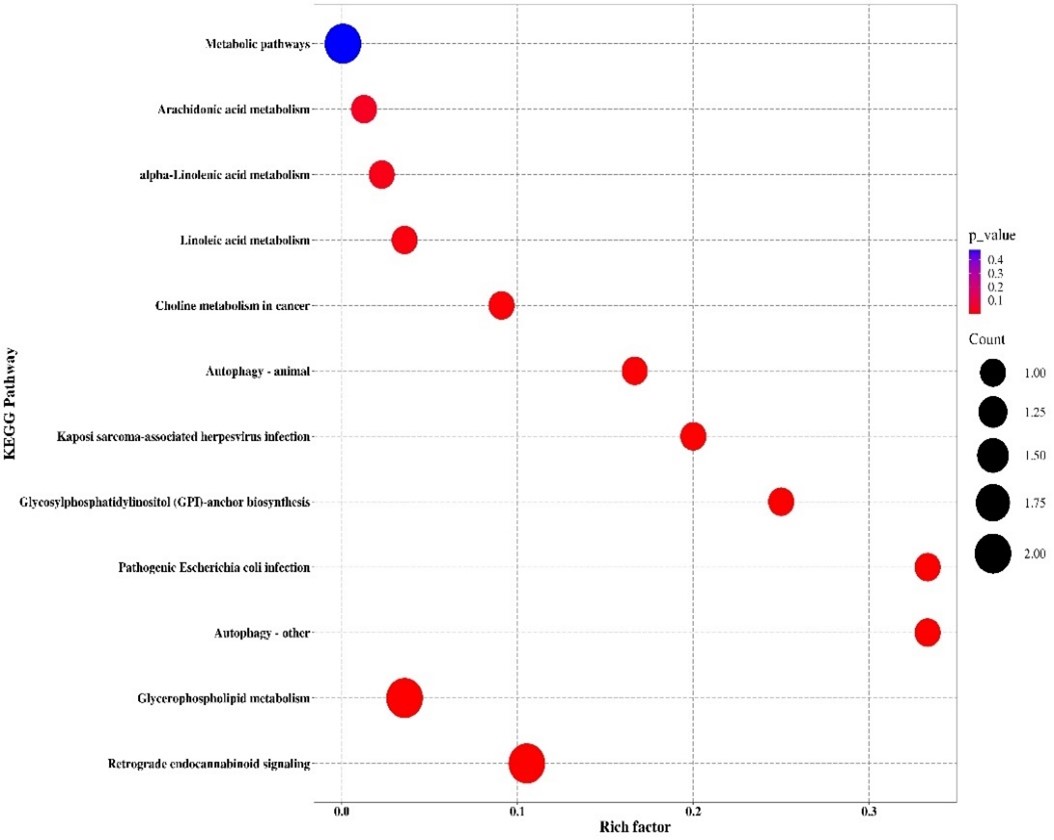
**

**Figure S2. Integrated analysis of lipidomic and transcriptomic data from Kazakh patients with ESCC.** A: Venn diagram showing the intersection of DEGs in the transcriptomic data and genes in the metabolite data. B: Pathways associated with lipidomic-related DEGs. C: Quantification of the FPKM levels of FADS1, ELOVL5, ACLY, ACOX2, and AMPKα2. N =27 * *P* < 0.05, ** *P* < 0.01.

**
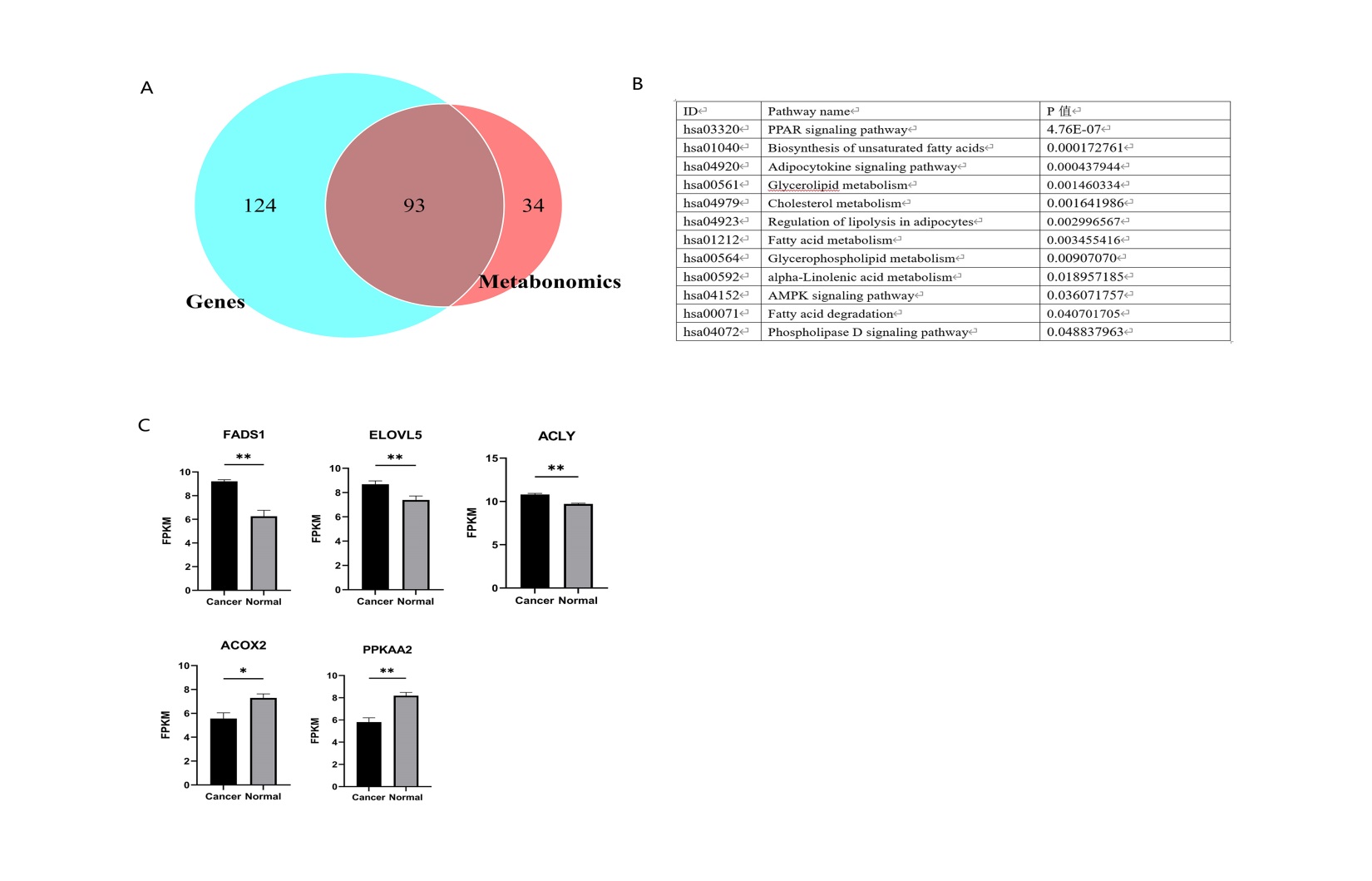
**

| **Table 1. Blood biochemical indexes of ESCC patients and healthy controls.** | | |
| --- | --- | --- |
| **Clinical index** | **ESCC group (n = 30, *‾χ*** *± s***)** | **Control group (n = 30, *‾χ*** *± s* |
| Age (years) | 61.45 ± 8.67 | 58.66 ± 5.93 |
| Sex (men/women, n) | 18/12 | 17/13 |
| Total cholesterol concentration (mmol/L) | 4.11 ± 0.65* | 6.10 ± 4.31 |
| Triglyceride concentration (mmol/L) | 0.98 ± 0.41 | 1.32 ± 0.82 |
| Low-density lipoprotein concentration (mmol/L) | 2.74 ± 0.74 | 3.09 ± 0.64 |
| High density lipoprotein concentration (-mol/L) | 1.26 ± 0.34 | 1.29 ± 0.24 |
| Total bilirubin concentration (mmol/L) | 11.48 ± 4.22 | 14.22 ± 7.02 |
| Total protein concentration (g/L) | 66.88 ± 5.62** | 71.40 ± 3.29 |
| Concentration of albumin (g/L) | 38.41 ± 4.16** | 44.42 ± 1.51 |
| Alkaline phosphatase concentration (U/L) | 81.86 ± 21.74 | 70.01 ± 19.16 |

**P* < 0.05, ***P* < 0.01, compared with the healthy control group.

**Table S1. General clinical data of ESCC patients and healthy subjects.**

| **Clinical characteristics** | **ESCC group(=30，*‾χ*** *± s***）** | **Control group(n=30，*‾χ*** *± s* **）** |
| --- | --- | --- |
| Age/(years） | 61.45 8.67 | 58.66 ± 5.93 |
| Sex (men/women, n) | 18/12 | 17/13 |
| Tumor invasion |  |  |
| T1-T2 | 13 |  |
| T3-T4 | 17 |  |
| Lymphatic metastasis |  |  |
| N0 | 21 |  |
| N1-2 | 9 |  |
| Differentiation degree |  |  |
| High-medium | 18 |  |
| Low | 12 |  |
| TNM stage |  |  |
| Ⅰ-Ⅱ | 16 |  |
| Ⅲ | 14 |  |
| Postoperative survival |  |  |
| ≥5(years） | 11 |  |
| ＜5（years） | 19 |  |

| **Table S2. The lipid internal standard.** | | |
| --- | --- | --- |
| **Actual concentration (mg/ml)** | **Dilution X** | **Internal standards** |
| 0.13 | 0.089285714 | CE (16:0)-IS |
| 0.13 | 0.089285714 | CE (16:1)-IS |
| 0.57 | 0.089285714 | CE (18:1)-IS |
| 1.43 | 0.089285714 | CE (18:2)-IS |
| 0.15 | 0.089285714 | CE (20:3)-IS |
| 0.18 | 0.089285714 | CE (20:4)-IS |
| 0.18 | 0.089285714 | CE (20:5)-IS |
| 0.22 | 0.089285714 | CE (22:6)-IS |
| 0.004 | 0.089285714 | Cer (18:0/16:0)-IS |
| 0.02 | 0.089285714 | Cer (18:1/16:0)-IS |
| 0.004 | 0.089285714 | DAG (16:0/16:0)-IS |
| 0.005 | 0.089285714 | DAG (16:0/18:0)-IS |
| 0.006 | 0.089285714 | DAG (16:0/18:1)-IS |
| 0.005 | 0.089285714 | DAG (16:0/18:2)-IS |
| 0.00135 | 0.089285714 | DAG (16:0/18:3)-IS |
| 0.0015 | 0.089285714 | DAG (16:0/20:4)-IS |
| 0.00145 | 0.089285714 | DAG (16:0/20:5)-IS |
| 0.016 | 0.089285714 | DAG (16:0/22:6)-IS |
| 0.005 | 0.089285714 | DAG (15:0/18:1)-IS |
| 0.05 | 0.089285714 | FFA (16:0)-IS |
| 0.05 | 0.089285714 | FFA (17:1)-IS |
| 0.03 | 0.089285714 | HexCer (18:1/16:0)-IS |
| 0.03 | 0.089285714 | Hex2Cer (18:1/16:0)-IS |
| 0.008522727 | 0.089285714 | LPC (16:0)-IS |
| 0.0125 | 0.089285714 | LPC (18:1)-IS |
| 0.05 | 0.089285714 | LPE (18:0)-IS |
| 0.08 | 0.089285714 | PC (15:0/18:1)-IS |
| 0.0025 | 0.089285714 | PE (15:0/18:1)-IS |
| 0.1 | 0.017857143 | SM (16:0)-IS |
| 0.1 | 0.017857143 | SM (18:1)-IS |
| 0.1 | 0.017857143 | SM (24:0)-IS |
| 0.1 | 0.017857143 | SM (24:1)-IS |
| 0.13 | 0.089285714 | TAG (50:1/FA16:0)-IS |
| 0.14 | 0.089285714 | TAG (52:1/FA18:0)-IS |
| 0.14 | 0.089285714 | TAG (52:2/FA18:1)-IS |
| 0.14 | 0.089285714 | TAG (52:3/FA18:2)-IS |
| 0.04 | 0.089285714 | TAG (52:4/FA18:3)-IS |
| 0.04 | 0.089285714 | TAG (54:4/FA20:3)-IS |
| 0.04 | 0.089285714 | TAG (54:5/FA20:4)-IS |
| 0.038 | 0.089285714 | TAG (56:7/FA22:6)-IS |
| 0.0275 | 0.089285714 | TAG (48:1/FA18:1)-IS |

| **Table S3. Primer information.** | |
| --- | --- |
| **Gene** | **Sequence (5'-3')** |
| hum-GAPDH-F | GGTCGGAGTCAACGGATTTG |
| hum-GAPDH-R | GGAAGATGGTGATGGGATTTC |
| ACLY-F | AACTTTTCTCTCCGCTCTG |
| ACLY-R | CCCTTCTGGTCCGCCTTCTTG |
| ACC1-F | ACCTGCGAGTAGAGACACAATTCC |
| ACC1-R | TCTTGGTGACTTGAGCGTGAGAG |
| CPT1-F | CGCTCGTGCTCATCTCCTACTG |
| CPT1-R | AGTTTGTTCTTCTGGCTTGTTTCCC |
| SCD1-F | ATACCACCACCACCACCATTACAG |
| SCD1-R | AGGGCGAATGTCGTCTTCCAAG |
| FADS1-F | GCAACTGGTTTGTGTGGGTGAC |
| FADS1-R | ATTGAAGGCAGACTTGTGGACATTG |
| ACOX2-F | CCCAGTGACCCAGAGGCAAAG |
| ACOX2-R | TGGTTCAGAATGGCAGTGTAGGAG |
| ELOVL5-F | GCACATTCCCTCTTGGTTGGTTG |
| ELOVL5-R | GTGGTCCTTCAGGTGGTCTTTCC |
| PPAR-F | CATCCCAGGCTTCGCAAACTTG |
| PPAR-R | CAGCATCCCGTCTTTGTTCATCAC |
| SREBP1-F | CACTGGTCGTAGATGCGGAGAAG |
| SREBP1-R | TTGTCATTGATGGAGGAGCGGTAG |
| HMGCR-F | CCAACCTACTACCTCAGCAAGCC |
| HMGCR-R | CCAGCCATTACGGTCCCACAC |
